# CyberKnife for hilar lung tumors: report of clinical response and toxicity

**DOI:** 10.1186/1756-8722-3-39

**Published:** 2010-10-22

**Authors:** Keith Unger, Andrew Ju, Eric Oermann, Simeng Suy, Xia Yu, Saloomeh Vahdat, Deepa Subramaniam, K William Harter, Sean P Collins, Anatoly Dritschilo, Eric Anderson, Brian T Collins

**Affiliations:** 1 Department of Radiation Medicine, Georgetown University Hospital, Washington, DC, USA; 2 Division of Hematology and Oncology, Georgetown University Hospital, Washington, DC, USA; 3 Division of Pulmonary, Critical Care and Sleep Medicine, Georgetown University Hospital, Washington, DC, USA; 4 Department of Pathology, Georgetown University Hospital, Washington, DC, USA

## Abstract

**Objective:**

To report clinical efficacy and toxicity of fractionated CyberKnife radiosurgery for the treatment of hilar lung tumors.

**Methods:**

Patients presenting with primary and metastatic hilar lung tumors, treated using the CyberKnife system with Synchrony fiducial tracking technology, were retrospectively reviewed. Hilar location was defined as abutting or invading a mainstem bronchus. Fiducial markers were implanted by conventional bronchoscopy within or adjacent to tumors to serve as targeting references. A prescribed dose of 30 to 40 Gy to the gross tumor volume (GTV) was delivered in 5 fractions. Clinical examination and PET/CT imaging were performed at 3 to 6-month follow-up intervals.

**Results:**

Twenty patients were accrued over a 4 year period. Three had primary hilar lung tumors and 17 had hilar lung metastases. The median GTV was 73 cc (range 23-324 cc). The median dose to the GTV was 35 Gy (range, 30 - 40 Gy), delivered in 5 fractions over 5 to 8 days (median, 6 days). The resulting mean maximum point doses delivered to the esophagus and mainstem bronchus were 25 Gy (range, 11 - 39 Gy) and 42 Gy (range, 30 - 49 Gy), respectively. Of the 17 evaluable patients with 3 - 6 month follow-up, 4 patients had a partial response and 13 patients had stable disease. AAT t a median follow-up of 10 months, the 1-year Kaplan-Meier local control and overall survival estimates were 63% and 54%, respectively. Toxicities included one patient experiencing grade II radiation esophagitis and one patient experiencing grade III radiation pneumonitis. One patient with gross endobronchial tumor within the mainstem bronchus developed a bronchial fistula and died after receiving a maximum bronchus dose of 49 Gy.

**Conclusion:**

CyberKnife radiosurgery is an effective palliative treatment option for hilar lung tumors, but local control is poor at one year. Maximum point doses to critical structures may be used as a guide for limiting toxicities. Preliminary results suggest that dose escalation alone is unlikely to enhance the therapeutic ratio of hilar lung tumors and novel approaches, such as further defining the patient population or employing the use of radiation sensitizers, should be investigated.

## Introduction

Patients presenting with inoperable lung tumors are generally treated with conventionally fractionated radiotherapy. To improve local control and survival, researchers in the past decade have explored various means of delivering high doses of radiation in shorter intervals [1]. Lung tumors have been treated with relatively tight margins (10 mm) utilizing a body frame and abdominal compression to restrict lung motion [2]. This enhanced precision has facilitated the safe delivery of highly effective hypofractionated doses of radiation quickly to peripheral lung tumors [3-16]. However, for central lung tumors, treatment related deaths have been attributed to radiation induced bronchial and esophageal injury [5,13]. An ongoing Radiation Therapy Oncology Group (RTOG) protocol is exploring potentially safer 5 fraction treatment regimens for small (< 5 cm) centrally located non-small cell lung cancers (NSCLCs) [17].

The CyberKnife^® ^System (Accuray Incorporated, Sunnyvale, CA) has been successfully employed at Georgetown University Hospital since early 2002 to treat stationary extracranial tumors [18]. With the introduction of the Synchrony™ motion tracking module in 2004, small peripheral and perihilar lung tumors that move with respiration have been successfully treated using tighter margins than previously feasible [19,20]. Here we report clinical results from 20 consecutive patients with hilar lung tumors abutting or invading the mainstem bronchus, treated in 5 fractions using the CyberKnife System with Synchrony™.

## Methods and Materials

### Eligibility

This retrospective review of an established departmental treatment policy was approved by the Georgetown University institutional review board. Consecutively treated patients between October 2005 and October 2009 with pathologically confirmed inoperable primary hilar lung cancers or hilar lung metastases were reviewed. A tumor was considered a "hilar lung tumor" if it abutted or invaded the mainstem bronchus. Baseline studies included PET/CT imaging with iodinated IV contrast as clinically feasible.

### Fiducial Placement

Tracking based on translational and rotational target information requires the use of a minimum of 3 non-collinear fiducials to be visible on the orthogonal images of the CyberKnife x-ray targeting system. Three to five gold fiducials measuring 0.8-1 mm in diameter by 3-7 mm in length (Item 351-1 Best Medical International, Inc., Springfield, VA) were placed in or near the tumors via bronchoscopy as previously described [21].

### Treatment Planning

Fine-cut (1.25 mm) treatment planning CT's were obtained following fiducial placement during a full inhalation breath hold with the patient in the supine treatment position. Gross tumor volumes (GTV) were contoured utilizing mediastinal windows. A treatment plan was generated using the TPS 5.2.1 non-isocentric, inverse-planning algorithm with tissue density heterogeneity corrections for lung based on an effective depth correction. The radiation dose was divided into 5 equal fractions of 6 to 8 Gy, prescribed to an isodose line that covered at least 95% of the planning treatment volume (PTV = GTV). Guidelines for dose limits to critical central thoracic structures are provided in Table 1. In general, prescribed doses were increased with clinical experience.

**Table 1:** Radiation maximum point dose limits

Adjacent Structure	Maximum Point Dose Limit (Gy) (total for 5 fractions)
Spinal cord	25

Left ventricle	30

Esophagus	40

Major bronchus	50

### Treatment Delivery

Patients were treated in the supine position with their arms at their sides. A form-fitting vest containing 3 red light-emitting surface markers was attached to the surface of the patient's anterior torso in the region of maximum chest and upper abdominal respiratory excursion. These markers projected to an adjustable camera array in the treatment room. Precise patient positioning was accomplished utilizing the automated patient positioning system. The internal fiducials were located using orthogonal x-ray images acquired with ceiling-mounted diagnostic x-ray sources and corresponding amorphous silicon image detectors secured to the floor on either side of the patient.

Prior to initiating treatment, an adaptive correlation model was created between the fiducial positions as periodically imaged by the x-ray targeting system and the light-emitting markers as continuously imaged by the camera array. During treatment delivery the tumor position was tracked using the live camera array signal and correlation model, and the linear accelerator was moved by the robotic arm in real time to maintain alignment with the tumor. Fiducials were imaged prior to the delivery of every third beam for treatment verification and to update the correlation model.

### Follow-up Studies

Patients were followed with physical examination and PET/CT imaging at 3 to 6 month intervals. Local tumor recurrence was defined as progression of the treated tumor on PET/CT imaging. Biopsies were obtained when clinically indicated. Early treatment response was defined by the Response Evaluation Criteria in Solid Tumors (RECIST) Committee [22]. Toxicities were scored according to the National Cancer Institute Common Terminology Criteria for Adverse Events, Version 3.0 [23].

### Statistical Analysis

Statistical analysis was performed with the MedCalc 11.1 statistical package. The follow-up duration was defined as the time from the date of completion of treatment to the last date of follow-up or the date of death. Actuarial local control and overall survival were calculated using the Kaplan-Meier method.

## Results

### Patient and Tumor Characteristics

Twenty consecutive patients (10 men and 10 women) were treated over a 4-year period (Table 2). Three patients presented with primary lung tumors (adenocarcinoma 1, squamous cell carcinoma 2) and 17 with hilar lung metastases (NSCLC 7, renal cell carcinoma 3, sarcoma 2, colon cancer 2, breast cancer 1, mesothelioma 1 and adenoid cystic cancer 1). The patients with primary lung cancer were treated with radiosurgery due to severe pulmonary dysfunction. The mean gross tumor volume (GTV) was 73 cc (range, 23 - 324 cc). Bronchoscopy for fiducial placement documented gross mainstem endobronchial tumor in 3 patients.

**Table 2:** Patient and Tumor Characteristics

Patient	Age	Sex	Performance Status (ECOG)	Symptom	Category	Histology	GTV (cc)	Mainstem Endobronchial Tumor
1	62	M	2	Cough	Metastasis	NSCLC	152	No

2	67	F	0	SOB	Metastasis	Sarcoma	179	No

3	79	M	2	SOB	Primary	NSCLC	137	No

4	71	F	2	Cough	Primary	NSCLC	221	No

5	65	F	2	SOB	Primary	NSCLC	68	No

6	13	M	0	None	Metastasis	Sarcoma	44	No

7	76	F	1	Cough	Metastasis	NSCLC	41	Yes

8	69	M	2	Pain	Metastasis	NSCLC	68	No

9	61	F	0	SOB	Metastasis	Renal	182	No

10	59	M	0	None	Metastasis	NSCLC	38	No

11	65	M	1	SOB	Metastasis	Mesothelioma	324	Yes

12	23	F	0	None	Metastasis	Colon	39	No

13	49	M	0	Cough	Metastasis	Renal	58	No

14	46	M	0	SOB	Metastasis	Colon	141	No

15	81	F	1	Cough	Metastasis	NSCLC	50	No

16	71	M	1	SOB	Metastasis	NSCLC	78	No

17	82	F	0	None	Metastasis	NSCLC	23	No

18	51	F	0	SOB	Metastasis	Breast	64	No

19	58	F	0	Cough	Metastasis	Salivary Gland	87	No

20	62	M	0	SOB	Metastasis	Renal	111	Yes

### Treatment Characteristics

Treatment plans were composed of hundreds of pencil beams delivered using a single 20 to 40-mm diameter collimator (median, 30 mm). Radiation was delivered in 5 equal fractions of 6 to 8 Gy each to a median prescription isodose line of 76% (range, 70-80%). The median dose delivered to the prescription isodose line over an average of 6 days (range, 5-8) was 35 Gy (range, 30-40 Gy). The resulting mean maximum point doses delivered to the esophagus and mainstem bronchus were 25 Gy (range, 11 - 39 Gy) and 42 Gy (range, 30 - 49 Gy), respectively.

### Early Clinical and Radiographic Response

**Figure 1:**
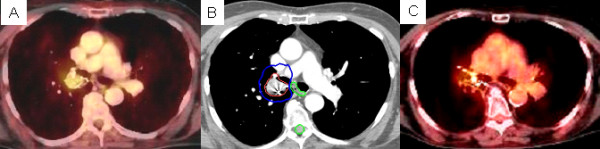
**Right hilar metastasis treatment planning PET/CT with a tumor SUV_max _of 9.6 (A), planned radiation dose distribution (B: the planning treatment volume is shown in red and the 35 Gy isodose line in blue), and PET/CT at 6 months post-treatment (C) shows an excellent response with a tumor SUV_max _of 2.7**.

All patients underwent clinical follow-up, and 14 patients reported symptomatic relief within 1 month of treatment and 2 patients reported relief by 4 months. Of the 17 patients with early radiographic follow-up, 4 patients experienced partial responses and 13 patients had stable disease at 3 - 6 months. There was no local disease progression within the 6-month follow-up interval. Furthermore, 13 patients with serial PET/CT imaging exhibited early declines in the maximum standardized uptake values (Figure 1).

### Local Control and Survival

**Figure 2:**
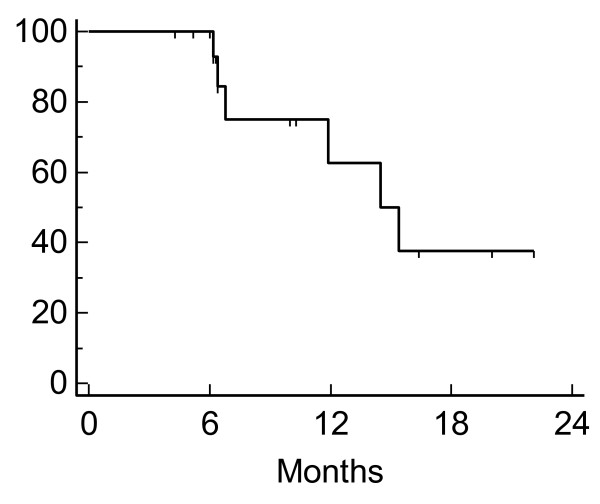
**Kaplan-Meier plot of local control**.

**Figure 3:**
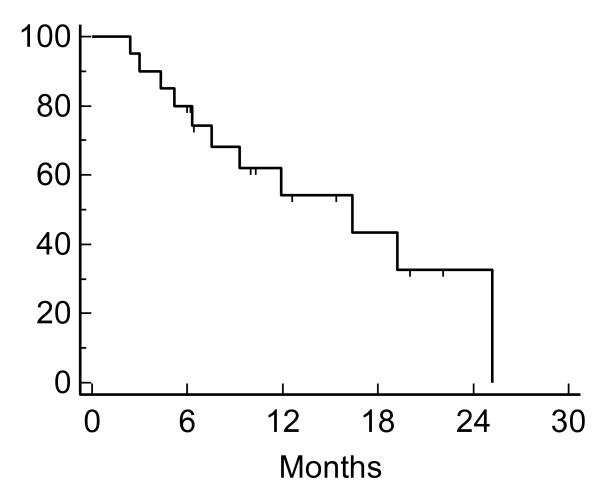
**Kaplan-Meier plot of overall survival**.

**Figure 4:**
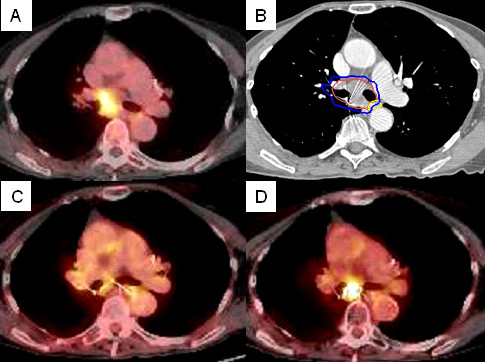
**Right hilar tumor treatment planning PET/CT with a tumor SUV_max _of 7.0 (A), planned radiation dose distribution (B: the planning treatment volume is shown in red and the 30 Gy isodose line in blue), and PET/CT at 6, and 12 months post-treatment (C and D) show an initial decrease in SUV_max _to 2.5 followed by local recurrence (SUV_max _= 7.2)**.

Despite excellent early clinical and radiographic responses, local control and survival outcomes beyond 6 months were poor. At a median follow-up of 10 months, the 1-year Kaplan-Meier local control and overall survival estimates were only 63% and 54%, respectively (Figure 2, 3). Deaths were largely attributed to metastatic disease (Table 3). However, despite limited follow-up and poor survival, 6 local failures were documented. One such failure resulted in a patient's death (Figure 4).

**Table 3:** Clinical Outcomes

Patient	Vital Status	Survival (Months)	Local Failure (Months)	Cause of Death
1	Dead	3	N/A	Metastases

2	Dead	4	N/A	Metastases

3	Dead	5	N/A	Metastases

4	Dead	6	N/A	Pulmonary

5	Dead	12	8	Metastases

6	Dead	19	12	Metastases

7	Dead	25	14	Metastases

8	Dead	9	8	Local Failure

9	Alive	N/A	N/A	N/A

10	Alive	N/A	N/A	N/A

11	Dead	7	N/A	Fistula

12	Alive	N/A	N/A	N/A

13	Dead	16	N/A	Metastases

14	Alive	N/A	15	N/A

15	Alive	N/A	9	N/A

16	Alive	N/A	N/A	N/A

17	Alive	N/A	N/A	N/A

18	Alive	N/A	N/A	N/A

19	Alive	N/A	N/A	N/A

20	Dead	3	N/A	Metastases

### Complications

**Figure 5:**
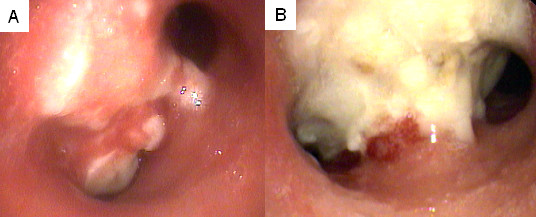
**Mainstem gross endobronchial tumor prior to treatment (A) and biopsy proven mainstem bronchus tumor necrosis at 7 months (B)**.

Strict maximum point dose constraints were maintained for normal tissues. Immediately following treatment, mild brief fatigue was reported by the majority of patients. Acute Grade II esophagitis, requiring brief hospitalization for IV hydration, was observed in 1 patient with renal cell carcinoma presenting with a relatively large GTV (182 cm^3^) and a high maximum esophageal point dose approaching the limit of 40 Gy. A second patient with severe COPD and progressing metastatic NSCLC developed dyspnea and an infiltrate on CT corresponding to the high dose treatment volume 8 months following CyberKnife treatment (40 Gy). He required temporary supplemental oxygen and his symptoms resolved with conservative treatment over a 4 day hospital stay. Finally, a patient with advanced mesothelioma developed a mainstem bronchus fistula 7 months following treatment and died (Figure 5). He was one of 3 patients with gross mainstem endobronchial disease. Additionally, the GTV was relatively large (324 cm^3^) and the mainstem bronchus received a maximum point dose of 49 Gy.

## Discussion

Continuous tracking of respiratory tumor motion and precise beam alignment throughout treatment permits greater dose conformality to the tumor contour and a sharp dose gradient [19,24]. We observed prompt symptomatic relief in 16 patients, likely due to the high dose per fraction. Furthermore, within 6 months of treatment there was no evidence of local tumor progression and the local control rate at 1 year was 63%. Our results compare favorably to a large RTOG trial of conventionally fractionated radiation therapy for palliation of inoperable NSCLC, which demonstrated palliation of symptoms in 60% and local control in 41% [25]. We conclude that stereotactic radiosurgery with real-time tumor motion tracking and continuous beam correction provides a well-tolerated and effective treatment option for hilar lung tumors.

Prior to proceeding with our institutional study of CyberKnife radiosurgery for hilar lung tumors, maturing data of others suggested that critical central thoracic structures tolerate high-dose hypofractionated radiation poorly [5]. In a phase II trial using 60-66 Gy in 3 fractions for the treatment of NSCLC, severe toxicity was noted in 46% of patients with central lung tumors at 2 years [5]. Therefore, we limited doses to 30-40 Gy in 5 fractions prescribed to the gross tumor volume without additional margin. In the absence of validated esophagus and mainstem bronchus dose limits for stereotactic radiosurgery in 5 fractions, we limited the maximum point doses to 40 Gy and 50 Gy, respectively. Although these dose limits were not exceeded, one patient developed grade II esophagitis and a second patient developed Grade III pneumonitis. Finally, one patient with gross mainstem endobronchial disease developed a fatal airway complication after receiving a maximum point dose of 49 Gy to the mainstem bronchus. In a recently published trial, 6 patients with lung tumors directly involving major airways (i.e. main or lobar bronchi) received 40 to 48 Gy in 4 fractions [13]. As with our study, treatment related toxicity was observed, including 3 patients who developed severe pulmonary toxicity. A single patient with gross mainstem endobronchial disease, who had received 48 Gy in 4 fractions, died of complication related to radiosurgery without evidence of tumor recurrence.

Despite the short survival of treated patients and the aggressive radiation doses used, local control at 1 year was a disappointing 63%. However, in light of dose limiting major bronchus, lung, and esophageal toxicity, further dose escalation beyond 40 Gy is not a feasible approach to improve local control in hilar tumors with a significant endobronchial component. Additional clinical trials that exclude patients with gross mainstem endobronchial disease will be necessary to define the appropriate patient characteristics and doses. Alternatively, this study provides support for investigation of novel radiation sensitizers to enhance the therapeutic ratio of hilar lung tumor radiosurgery.

## Conclusion

Hilar lung tumor patients may be treated with frameless stereotactic radiosurgery, resulting in encouraging early clinical responses, acceptable acute toxicity and reliable palliation. However, local control at 1 year remains poor despite aggressive radiation doses and life threatening late toxicity has been reported, especially for tumors with a significant endobronchial component. We propose additional clinical investigation optimizing patient selection and consideration of novel combination treatments with radiation sensitizing drugs.

## List of Abbreviations

CT: computed tomography; GTV: gross tumor volume; GY: Gray; NSCLC: non-small cell lung cancer; PET: positron emission tomography; PTV: planning treatment volume; and SUV_MAX_: maximum standardized uptake value;

## Competing interests

BC is an Accuray clinical consultant. EA is paid by Accuray to give lectures.

## Authors' contributions

KU participated in data collection, data analysis and manuscript drafting and manuscript revision. AJ participated in data collection, data analysis and manuscript revision. EO participated in data collection, data analysis and manuscript revision. SS created tables and figures and participated in data analysis and manuscript revision. XY participated in treatment planning, data collection and data analysis. SV participated in data collection, data analysis and manuscript revision. DS participated in data analysis and manuscript revision. KWH participated in treatment planning, data analysis and manuscript revision. SC prepared the manuscript for submission, participated in treatment planning, data collection, data analysis and manuscript revision. AD participated in data analysis and manuscript revision. EA participated in treatment planning, data collection, data analysis and manuscript revision. BC drafted the manuscript, participated in treatment planning, data collection and data analysis. All authors have read and approved the final manuscript.
